# A Longitudinal Study of Long-Term Change in Contamination Hazards and Shallow Well Quality in Two Neighbourhoods of Kisumu, Kenya

**DOI:** 10.3390/ijerph120404275

**Published:** 2015-04-17

**Authors:** Joseph Okotto-Okotto, Lorna Okotto, Heather Price, Steve Pedley, Jim Wright

**Affiliations:** 1 Victoria Institute for Research on Environment and Development (VIRED), International Rabuour Environment and Development Centre, Kisumu-Nairobi Road, P.O. Box 6423-40103, Kisumu, Kenya; E-Mail: jokotto@hotmail.com; 2 School of Spatial Planning and Natural Resource Management, Jaramogi Oginga Odinga University of Science and Technology, Bondo (Main) Campus, P.O. Box 210-40601, Bondo, Kenya; E-Mail: lgokotto@yahoo.com; 3 Geography and Environment, University of Southampton, Highfield Campus, Southampton SO17 1BJ, UK; E-Mail: h.price@soton.ac.uk; 4 Robens Centre for Public and Environmental Health, University of Surrey, Guildford, Surrey GU2 7XH, UK; E-Mail: s.pedley@surrey.ac.uk

**Keywords:** groundwater, water pollution, water wells, urbanization, latrines, longitudinal survey

## Abstract

Sub-Saharan Africa is experiencing rapid urbanisation and many urban residents use groundwater where piped supplies are intermittent or unavailable. This study aimed to investigate long-term changes in groundwater contamination hazards and hand-dug well water quality in two informal settlements in Kisumu city, Kenya. Buildings, pit latrines, and wells were mapped in 1999 and 2013–2014. Sanitary risk inspection and water quality testing were conducted at 51 hand-dug wells in 2002 to 2004 and 2014. Pit latrine density increased between 1999 and 2014, whilst sanitary risk scores for wells increased between 2002 to 2004 and 2014 (n = 37, Z = −1.98, *p* = 0.048). Nitrate levels dropped from 2004 to 2014 (n = 14, Z = −3.296, *p* = 0.001), but multivariate analysis suggested high rainfall in 2004 could account for this. Thermotolerant coliform counts dropped between 2004 and 2014, with this reduction significant in one settlement. Hand-dug wells had thus remained an important source of domestic water between 1999 and 2014, but contamination risks increased over this period. Water quality trends were complex, but nitrate levels were related to both sanitary risks and rainfall. Given widespread groundwater use by the urban poor in sub-Saharan Africa, the study protocol could be further refined to monitor contamination in hand-dug wells in similar settings.

## 1. Introduction

Urban Africa is expanding rapidly, with its population estimated to grow from around 400 million in 2010 to 1.2 billion by 2050 [1]. Over a quarter of the world’s 100 fastest-growing cities are now in Africa and this rapid growth raises concerns over water security. These water security concerns partly arise as utilities responsible for piped water and sewerage struggle to keep pace with population growth and demand for such services. As a consequence, in unplanned settlements residents often either rely on vendors selling piped water, illegal connections to the water distribution system, or use groundwater sources where piped supplies are inadequate or intermittent [2].

In many cities in Sub-Saharan Africa, shallow wells are a widespread form of self-supply to obtain domestic water, and their use in many expanding urban centres has been documented [3]. Although they are relatively cheap to build and maintain where conditions are suitable [4], they are often at risk of contamination due to a number of factors, including inflow from surface runoff, contamination as water is abstracted by users, and/or contaminant transport through the unsaturated and saturated zones. As unplanned urban development takes place and population density increases, pit latrines, open defecation by both humans and livestock, uncollected waste and other contamination hazards may potentially develop close to groundwater sources that were originally constructed on sites some distance from such hazards. Well water contamination in these environments therefore can be associated with inadequate well lining and protection of the wellhead, by-pass pathways such as contaminated buckets and ropes, but also by ingress of faecal matter into the underlying aquifer from surrounding hazards such as pit latrines [5]. 

Understanding the long-term changes in domestic water access and use can provide insights into sustainability and long-term trends that may continue into the future. For example, the Drawers of Water II study [6] surveyed 1015 urban and rural households in Kenya, Uganda, and Tanzania in 1997–2000 within the same communities as the original Drawers of Water I study, which took place 30 years previously [7]. In the Drawers of Water II study, it was found that the quantity of piped water consumed per capita had declined in the intervening years, whereas the quantity of unpiped water consumed per capita had increased [6]. This trend reflected declining reliability in piped water supplies and the growth of an urban and peri-urban population without access to piped water. As such, this type of study can help inform future planning of water supply and safety. Long-term change studies can also provide stronger evidence of causality than would be apparent in cross-sectional data collected at a single point in time. Although there are many cross-sectional studies of drinking-water supply and use, there are far fewer long-term studies. 

Monitoring the long-term change in groundwater quality is generally focused on deep aquifers, where typically there is a lag of years or decades between the ingress of contaminants from surface hazards and its impact on groundwater quality. For example, examination of long-term data for a drilled well field in Mochudi, Botswana suggest nitrate levels rose from <1 mg/L in the 1960s to over 30 mg/L in the 1980s [8], whilst borehole data for Bamako, Mali suggest median nitrate as total N rose from 5 mg/L in 1990 to over 20 mg/L by 2002 [9]. However, whilst deep aquifers are an important potential source of domestic water, boreholes and deep wells are expensive to construct and are only affordable by wealthy individuals or accessible to the urban poor through donor funded projects. In view of their modest construction costs, shallow hand-dug wells are far more widely used by the urban poor in sub-Saharan Africa. From a public health and population exposure standpoint, it is therefore the contamination of shallow aquifers that poses the greatest potential hazard to human populations. Such shallow aquifers respond more rapidly to the changing distribution of hazards such as onsite sanitation, broken sewerage pipes, and uncollected waste at the surface. Simultaneously, short-term water quality fluctuations in shallow aquifers also make it more difficult to identify longer term water quality trends.

The purpose of this study is to examine long-term changes in shallow well water contamination hazards within the built environment and well water quality in two settlements with unplanned development of residential buildings in Manyatta and Migosi in Kisumu, Kenya.

## 2. Experimental Section 

### 2.1. Study Area 

**Figure 1:**
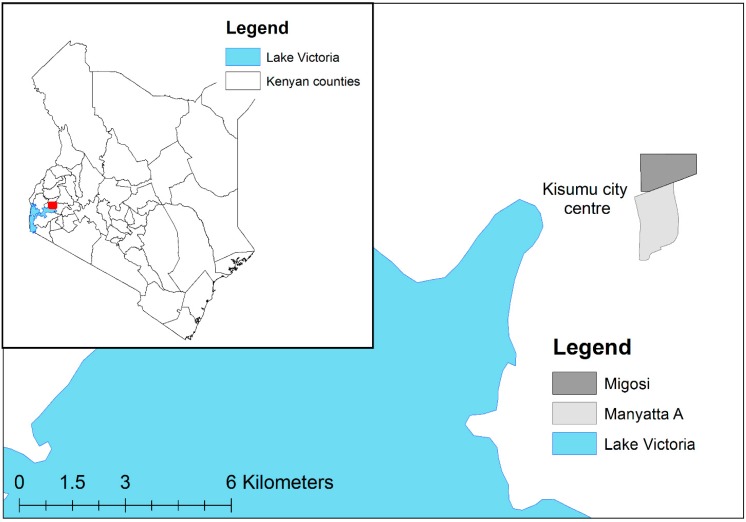
Location of Manyatta A and Migosi in Kisumu, Kenya.

The study area is located in Kisumu City in the County of Kisumu, western Kenya. Kisumu City is situated on the eastern extremities of Lake Victoria on the shores of Winam Gulf. The city area extends between latitude 00°02′N; 00°11′S and longitude 34°35′; 34°55′E, at an elevation of approximately 1174 m above sea level. The total area covered by the city is approximately 417 km^2^ of which 35.5% comprises the waters of Lake Victoria. With an estimated urban core population of 259,258 in 2009 [10], Kisumu is Kenya’s third largest city. The city was originally the terminus of the railway from Mombasa, and consisted of a core European residential area surrounded by higher density unplanned settlements intended for the African native populations [11]. Two of these unplanned settlements, namely Migosi and Manyatta A to the east of Kisumu’s city centre (Figure 1), form the focus of the current study. 

Migosi and Manyatta cover an area of 1.93 km^2^ and 2.36 km^2^, respectively. According to the 2009 census, the population densities in Manyatta A (a sublocation of Manyatta) and Migosi was 203 and 103 people per Ha, respectively. Thirty nine percent of households in Manyatta A and 24% of households in Migosi were using groundwater as their main source of domestic water, with 31% and 23%, respectively, using piped water. A further 29% of households in Manyatta A and 50% in Migosi purchased water from vendors, some of which may have originated from groundwater. Ninety one percent of Manyatta A and 38% of Migosi households used pit latrines, with 4% and 29%, respectively, using septic tanks. Open defecation is practiced by less than 1%. Mains sewerage was widespread in Migosi (32%) but rare in Manyatta A (5%).

The geological formations underlying the gently sloping landscape in the study area are tertiary phonolitic lavas. Partly decomposed phonolites and a few porphyritic granite intrusions dating back to the post-Nyanzian and Bukoban periods are exposed in places [12]. The exposed rock material reveals bouldered rock formations of varying sizes with extensive fractures and joints particularly towards the surface. A pyroclastic deposit overlies the phonolites and this increases in thickness towards the southeast of both study sites. Wells in the west are thus dug into the fractured phonolitic basalt, whilst those to the east are located in pyroclastics. A relatively thin layer of reddish-brown soil generally covers the rock surface. The lateritic and decomposed rock material that forms the basis of the soil associations in the area is of medium to low plasticity and is well drained in most places [13]. There is a small section of the low lying eastern areas of Migosi where drainage seems to be constrained. This leads to occasional water logging in such areas and a few of the wells (about 2%) are often inundated during the wet season. The shallow well depth, fracturing, and lack of thick soil cover in the study area result in a highly vulnerable aquifer system.

### 2.2. Monitoring of Rainfall and Contamination Hazards in the Built Environment

Building outlines were digitised in both Manyatta A and Migosi in 1998 and again in 2013. Buildings were identified on 1:2500 and 1:5000 mosaicked basemaps produced in 1989 and updated from 1:2500 aerial photography for the year 1998. Subsequently, a high spatial resolution WorldView-II satellite image was acquired from September 2013 and georeferenced using the 1998 building outlines. Buildings visible on the 2013 imagery were then digitised through visual interpretation. Building density was then calculated for both the 1999 baseline year and for 2013.

The 1998 mosaicked aerial photography was also used as the basis for developing map layers depicting the locations of wells and pit latrines. The data were supplemented by a follow-up rapid mapping exercise in 1999 in which every compound in the study area was visited and water and sanitation facilities identified (see a previous study for further details [14]). In August 2014, a transect survey was used to update the 1999 baseline map layers of pit latrines and wells. Two 100 metre wide transects were surveyed across the study area between randomly generated start and end points. The transect through Migosi was 1.7 km long, whilst that in Manyatta A was 2.4 km long. Within each transect belt the locations of shallow wells and pit latrines were surveyed and any changes noted in comparison to the 1999 1:2500 basemaps.

In earlier work, a sample of 51 shallow wells was selected from the 1999 map and visited between 2002 and 2004 [14]. Sanitary risk inspections were undertaken at each source using a standard, structured observation checklist [15] that identified 15 contamination risks either in the surrounding environment (e.g., pit latrines or uncollected waste) or at the well itself (e.g., inadequate well shaft lining or absence of a well cover). The same sample of wells was revisited in March–April 2014 and for those still accessible, the same checklist was used to assess contamination risks in or around each well. The individual checklist items were combined into a single overall percentage risk score, based on the proportion of items where a risk was present, with higher scores indicating a greater risk of contamination.

Census data were acquired for 2009, 1999, and 1989, but temporal comparisons proved infeasible because of changes in both sub-location boundaries and question wording between successive censuses. Daily gridded rainfall estimates with a spatial resolution of 0.1 degrees (approximately 6 × 6 km) were downloaded from http://earlywarning.usgs.gov/fews/index.php for each day of sampling and the preceding three days. We examined rainfall patterns within the preceding 3 days only because of some evidence from Mozambique to suggest shallow well quality responded rapidly to rainfall onset [16]. These data are derived from a combination of precipitation data from approximately 1000 meteorological stations combined with satellite imagery from three sources that detects cold cloud tops associated with convective precipitation [17].

### 2.3. Monitoring of Well Water Quality 

Drawing on the 1999 basemap, a sample of 51 wells was tested for thermotolerant coliforms (TTCs) and electroconductivity on an ongoing basis between 2001 and 2004. 80% of 207 samples collected from these wells were taken in February to May, with the remainder taken in June, July and November. These wells were re-tested between February and March 2014. In both periods, an approximately 500 mL sample of water was collected from each well using a stainless steel sampling cup that had been disinfected with 70% v/v ethanol and then rinsed twice with well-water. The water samples were transferred into sterile plastic bottles and then stored in iced cooler boxes, in the dark, until they reached the laboratory. All samples were processed within six hours of collection. 

In both the baseline and follow-up studies, TTCs were isolated and quantified using the membrane filtration method with Membrane Lauryl Sulphate Broth (MLSB, Oxoid, Basingstoke, UK) as the selective medium. Two volumes of each sample—normally 1 mL and 10 mL—were filtered under vacuum pressure through a 0.45 µm nitrocellulose gridded membrane filter (Pall-Gelman, Portsmouth, UK). The membranes were transferred aseptically to pads saturated with MLSB, pre-incubated at room temperature for one hour and then transferred to a 44 ± 0.5 °C incubator (DelAgua Water Test Kit, DelAgua Water Testing Ltd., Lower Fyfield, Wiltshire, UK) for a total incubation time of 20 ± 2 h. After incubation, the membranes were removed from the incubator and examined within 15 min for typical coliform colonies (cream to yellow colonies, greater than 1 mm diameter). The Delagua Water Test Kit has a limited capacity that did not allow for regular replicate analysis of samples and the determination of analytical precision by statistical methods. Consistency of analysis was maintained by using the same analyst for all samples in the baseline and the follow-up studies. Precision was monitored by comparing the calculated density of TTC per 100 mL from the two sample volumes when both samples were in a countable range. The number of colonies was recorded and then standardised to 100 mL of the original sample. No further confirmation tests were done on the isolates. Conductivity was measured using a COND3110 handheld conductivity meter calibrated with appropriate standard solutions provided by the manufacturer.

Since elevated nitrate and chloride levels are often associated with faecal contamination, during November 2004, nitrate, sulphate and chloride concentrations were measured at 18 of the sampled wells using a Palintest photometer, model 5000, with dedicated reagents (Palintest Ltd., Gateshead, UK) and following the manufacturer’s instructions. In February–March 2014, the 51 wells (including the sub-sample of 18) were revisited to re-test samples for nitrate, sulphate and chloride once more as well as phosphate. A Palintest photometer model 7100 was used during this follow-up survey. The depth to the water in the wells, and the depth of the water, was measured using a dip metre.

### 2.4. Statistical Analysis 

Testing for statistically significant change in well water and environmental parameters over time was undertaken in SPSS (version 21, IBM UK Ltd, Portsmouth, UK) using the non-parametric Wilcoxon signed-rank test. Microbiological data were log10 transformed prior to all statistical analyses. To account for samples with undetectably large or small TTC counts [18], interval regression was used in Stata (version 11, Stata Corporation, College Station, TX, USA) to estimate geometric mean indicator bacteria counts per 100 mL. Interval regression was also used to examine the relationship between log TTC counts and sanitary risk scores, rainfall immediately preceding sample collection, building density, and time elapsed since the start of the study. Linear regression was used to examine the relationship between nitrate levels and sanitary risk scores, rainfall, building density and whether samples were collected in the baseline or follow-up periods.

Preliminary findings were presented to members of both communities and representatives of community-based organisations to gain further insights into patterns of change. Ethical approval for the study was obtained from the Faculty of Social and Human Sciences, University of Southampton (reference: 8350) and the University of Surrey (EC/2014/19/FEPS).

## 3. Results 

### 3.1. Follow-Up Survey of Hand-Dug Wells 

**Figure 2:**
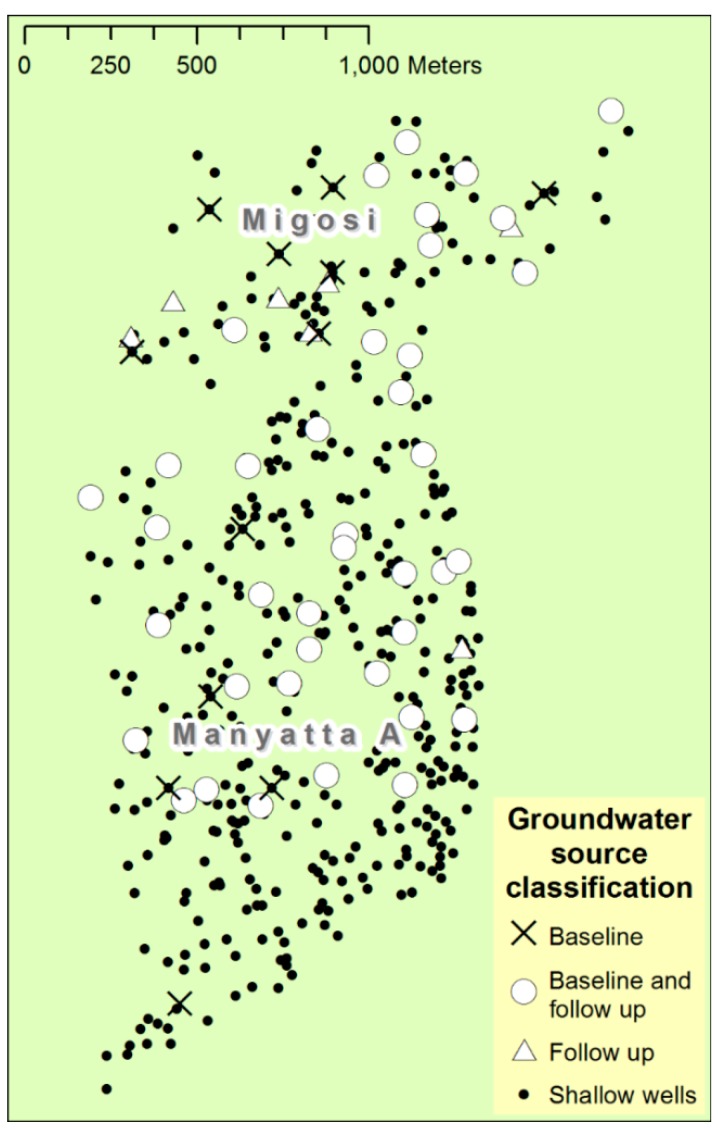
Map showing the locations of wells in Manyatta A and Migosi sampled during the baseline (1998–2004) and follow-up surveys (2014).

Figure 2 shows the distribution of wells in Manyatta A and Migosi that were sampled in the baseline (1998–2004) and follow-up (2014) campaigns. Fifty one wells were included in the baseline survey and 46 wells in the follow-up survey. 

Of the 46 wells tested during the follow-up survey, 39 had been tested during the baseline survey. Of the seven wells included only in the follow-up survey but not in the baseline, four were nearest accessible neighbour replacements for wells that were included in the baseline survey but had since fallen into disuse. The other three wells were added to improve the spatial distribution of wells across Manyatta A and Migosi. There was a 23.5% loss (12 wells) of sampled wells between the baseline and follow-up campaigns. Of these 12 wells, six were built over or abandoned (e.g., due to sewage influx), one was inaccessible during sampling and five were not resampled since they were only sampled on one occasion during the 1998–2004 baseline campaign. 

Table 1 summarises the overall findings of the study in terms of well locations, water quality and potential risk factors in both the baseline and follow-up campaigns. Only 11 of 207 (5%) baseline samples and four of 46 (9%) follow-up samples had no detectable TTCs with all others being above the WHO guideline values. Ten of 18 baseline samples (56%) but only two of 46 follow-up samples (4%) were above the guideline value for nitrate.

**Table 1:** Summary of well and water quality parameters and selected potential contamination risk factors (arithmetic means unless otherwise stated).

Parameter	Baseline Mean (Range)	Follow-Up Mean (Range)	No. of Samples (Baseline)	No. of Samples (Follow-Up)
***Well parameters:***				
Number of sampled wells in Manyatta A	33	30	n/a	n/a
Number of sampled wells in Migosi	18	16	n/a	n/a
Depth to water (Manyatta A) in metres	NM	3.3 (1.7–4.9)	n/a	29 ^a^
Depth to water (Migosi) in metres	NM	2.7 (0.1–4.7)	n/a	16
Depth of water in well (Manyatta A) in metres	NM	1.3 (0.1–2.9)	n/a	29 ^a^
Depth of water in well (Migosi) in metres	NM	2.9 (0.4–11.2)	n/a	16
***Water quality (Manyatta A and Migosi combined):***				
Electro-conductivity (µS)	646 (0–2006)	809 (173–3525)	212 (from 51 wells)	46
Nitrate (NO_3_ as N; mg/L)	15.1 (0.06–45.0)	4.1 (0.08–20.2)	20	46
Chloride (mg/L)	80 (0–225)	67 (1–90)	20	46
Sulphate (mg/L)	39 (5–93)	45 (14–94)	20	46
Phosphate (mg/L)	NM	1.9 (0.4–17.5)	n/a	38 ^b^
Thermotolerant coliforms (cfu/100mL) ^c^	2183 (<10–>100,000)	1120 (<10–11,200)	207 (from 51 wells)	46
***Risk factors (Manyatta A and Migosi combined):***				
Mean sanitary risk score for wells (%)	69.6 (27.0–93.1)	76.8 (53.0–100.0)	47	46
Rainfall (mm)	0.1 (0–12)	2.6 (0–3)	46	46
% Area occupied by buildings—Manyatta A	17.6%	27.3%	n/a	n/a
% Area occupied by buildings—Migosi	10.4%	23.4%	n/a	n/a

**^a^** Depth not measurable at one covered well; **^b^** Eight samples could not be tested due to reagent expiration; **^c^** Geometric mean thermotolerant coliforms calculated using interval regression.

### 3.2. Assessment of Changes to Contamination Hazards in the Built Environment

Table 1 shows the change in building density between 1999 and 2013 in both settlements. In 1999, Migosi had a much lower building density than Manyatta A, but by 2013 the proportion of the planimetric area occupied by buildings in the two settlements had almost converged and had increased substantially in both cases. Table 2 shows the changes in the number of hand-dug wells and pit latrines since 1999, according to the transect survey of Manyatta A and Migosi. Although a small number of hand-dug wells and pit latrines had fallen into disuse in both Migosi and Manyatta A, these were outweighed by the larger number of new wells and latrines that had been constructed since 1999.

**Table 2:** Results of a 2014 transect survey of hand-dug wells and pit latrines in the neighbourhoods of Migosi and Manyatta A, Kisumu.

Neighbourhood	Hand-Dug Wells			Pit Latrines		
	Present in 1999 and 2014	Present in 1999 Only	Present in 2014 Only	Present in 1999 and 2014	Present in 1999 Only	Present in 2014 Only
Migosi	14	6	12	44	3	21
Manyatta A	46	3	24	209	9	40

**Figure 3:**
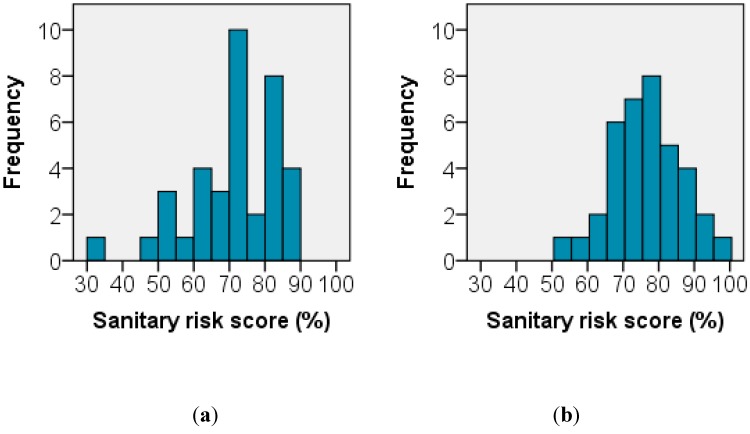
Histograms of sanitary risk scores calculated for paired wells (n = 37) in Manyatta A and Migosi during (**a**) the baseline survey (1999–2004) and; (**b**) the follow-up survey (2014).

Figure 3 shows the change in sanitary risk scores between 1999 and 2014 for the sample of hand-dug wells. Of the 39 matching wells between the baseline and follow-up, 37 wells had sanitary risk data in both surveys. For the 37 paired wells in Manyatta A and Migosi the mean sanitary risk score increased from 70.6% (standard deviation = 12.6%) in the baseline to 76.6% (standard deviation = 9.9%) in the follow-up survey. Between the baseline and the follow-up surveys there was a small shift from lower risk scores (between 30% and 50%) to the very high risk scores (90% to 100%). The increase in sanitary risk scores between baseline and follow-up was statistically significant (Wilcoxon signed-rank test, Z= −1.977, *p* value = 0.048, r = −0.23 (small effect size)).

### 3.3. Assessment of Changes to Well Water Quality

**Figure 4:**
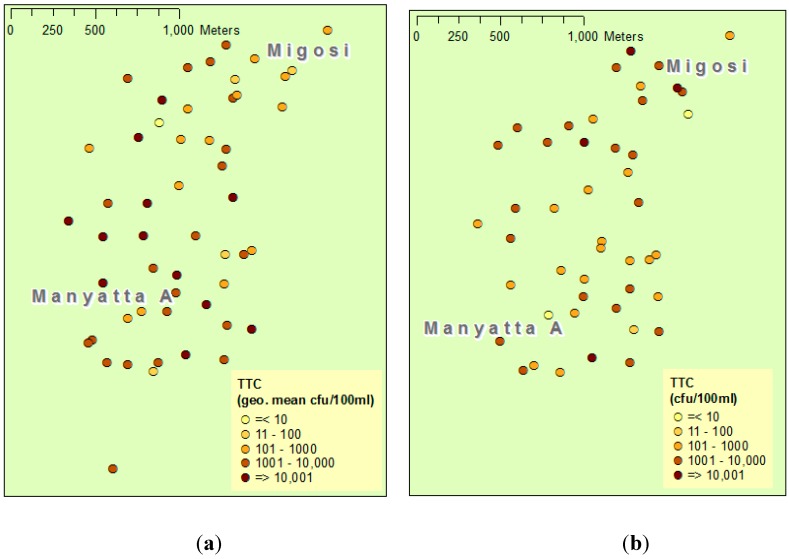

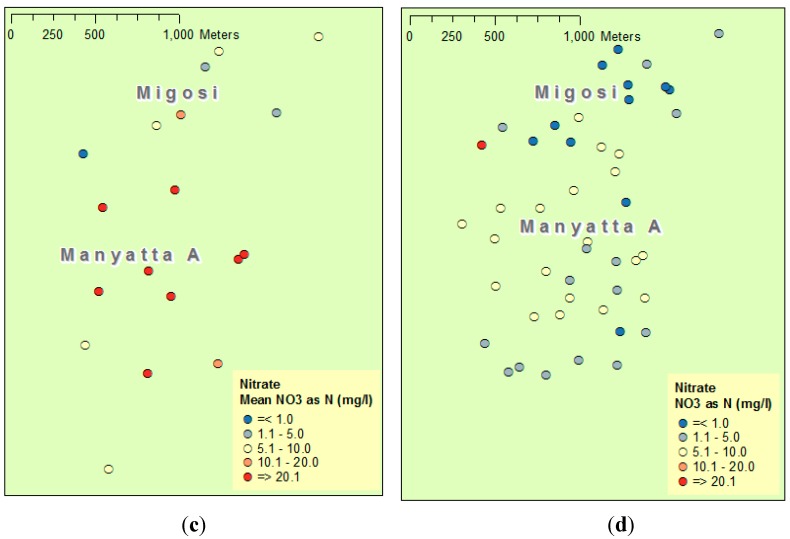
Maps of Manyatta A and Migosi showing pollutant concentrations in individual wells for: (**a**) Thermotolerant coliforms (TTC) 1998–2004 (geometric mean of all samples taken during this period); (**b**) TTC 2014; (**c**) Nitrate 2004; **(d)** Nitrate 2014.

Figure 4 shows the spatial distribution of nitrate concentration and TTC counts in shallow wells in the baseline and follow-up surveys. For nitrate, in both Manyatta A and Migosi, nitrate concentrations had reduced between baseline and follow-up surveys. From Figure 4, the TTC counts in samples from Manyatta A fell between surveys, while in Migosi the counts increased.

**Figure 5:**
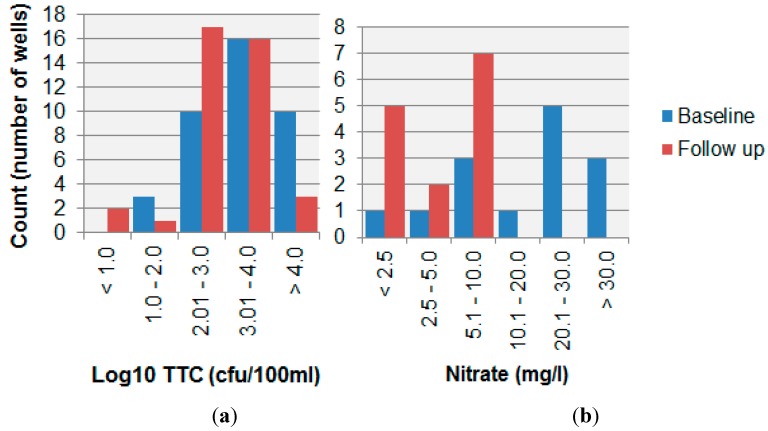
Comparison of water quality for pairs of wells tested in both baseline (1998–2004) and follow-up surveys (2014) for (**a**) thermotolerant coliforms (n = 39); (**b**) nitrate (n = 14).

There were 39 matched wells (baseline and follow-up) with TTC data (Figure 5). For matched wells, the median TTC count decreased from 2718 cfu/100 mL to 620 cfu/100 mL, and this reduction was statistically significant (Wilcoxon signed-rank test, Z= −2.226, *p* = 0.026, r= −0.252 (small effect size)). Of the 18 wells for which there were nitrate data from the baseline survey, 14 could be matched to wells in the follow-up survey. Three of the remaining wells fell into disuse and were replaced with a nearest neighbour in the follow-up survey and are therefore not directly comparable, and one was not re-sampled in the follow-up survey. For 14 paired wells, the mean nitrate concentration was 19.8 mg/L in the baseline survey (Mdn 24.0 mg/L) and 3.9 mg/L in the follow-up survey (Mdn 5.0 mg/L). This reduction in nitrate concentration between baseline and follow-up surveys was statistically significant (Wilcoxon signed-rank test, Z= −3.296, *p* = 0.001, r = −0.0623 (large effect size)). In contrast, a Wilcoxon signed-rank test on 39 paired samples suggested conductivity was significantly higher for shallow wells tested in 2014 (Mdn = 688 µS) compared to the baseline survey (Mdn = 652 µS), Z = −2.01, *p* = 0.44). 

Table 3 shows the coefficients of a linear regression model predicting nitrate levels from risk factors in samples, fitting a combined model to water quality and risk factor data from both baseline and follow-up. There was a positive relationship between both sanitary risk scores and preceding rainfall and nitrate levels. Table 3 also suggests that after accounting for the effect of recent rainfall and sanitary risk scores, baseline nitrate levels were significantly higher in Manyatta but lower in Migosi.

**Table 3:** Linear regression results, showing risk factors for nitrate contamination of hand-dug wells at baseline (2004) and in 2014 (n = 58, adjusted R^2^ = 0.78).

Covariate	Coefficient	Standard Error	T	*p*-Value
Sanitary risk %	0.11	0.05	2.17	0.04
Mean daily rainfall on day & preceding three days (mm)	1.41	0.36	3.95	0.00
Baseline measurement—both sites (binary)	−12.80	4.11	−3.12	0.00
Baseline measurement from Manyatta A (binary)	29.87	3.20	9.33	0.00
Constant	−5.60	4.17	−1.34	0.18

Table 4 shows the significant coefficients of an interval regression model predicting TTC counts in well samples from both 2002 to 2004 and 2014, based on rainfall, sanitary risk, period, and location. There was no apparent relationship between sanitary risk and TTC concentration and there was a weak inverse relationship between preceding rainfall and TTC contamination. After controlling for this relationship, TTC contamination was significantly higher in Manyatta at baseline only.

**Table 4:** Interval regression results, showing risk factors for thermotolerant coliform contamination of hand-dug wells at baseline (2002–2004) and in 2014 (n = 253, Wald chi squared = 26.9).

Covariate	Coefficient	Robust Standard Error	z	*p* value
Mean daily rainfall on day & preceding three days (mm)	−0.04	0.01	−3.64	<0.001
Baseline sample from Manyatta	0.55	0.11	4.83	<0.001
Constant	3.04	0.09	34.39	<0.001

## 4. Discussion 

With large urban population growth projected for Sub-Saharan Africa, a key issue in strategic planning is the way in which water and sanitation service access will evolve in response [1]. In our case study of two well-established neighbourhoods in Kisumu, the transect survey evidence (Table 2) suggests that the numbers of pit latrines and hand-dug wells have increased between 2002 and 2014, and that domestic use of shallow groundwater has persisted for over a decade, and probably longer. This local trend reflects a broader national reduction in the proportion of urban Kenyans using improved water sources from 87% in 2000 down to 82% by 2012 [19]. This increase has taken place at the same time as the formal water sector in the city has worked to expand the supply of piped water in these neighbourhoods—largely through standpipe sources—and alongside a growth of water-vending by individuals and organized groups. The much cheaper cost of well water relative to vended piped water in areas lacking piped water connections [20] is a likely explanation for this emerging scenario.

As evidenced by the increase in building density (Table 1), pit latrine density (Table 2), and the increase in sanitary risk (Figure 3), the potential for contamination appeared to have increased from 1999 to 2014. Despite these increasing hazards, nitrate levels apparently decreased (Figure 4 and Figure 5). However, in examining long-term change in shallow well water quality, a particular difficulty is the potential short-term impact of rainfall events. For example, a past study identified a noticeable effect of rainfall events on the quality of springs in Kampala [21], and another study reported an initial TTC spike during rainfall onset in shallow wells in Mozambique as contaminants were flushed into wells [16]. The emergence of daily precipitation data sets generated through a consistent procedure for each period [17] provides an opportunity to control for such events when analysing water quality. In our study, there was an apparent decrease in some parameters such as nitrate (Figure 4 and Figure 5). Were this to reflect contamination of the aquifer and not contamination via by-pass pathways such as buckets and ropes or contaminant ingress via the wellhead, this apparent decrease would run counter to the nitrate “time-bomb” [22] often observed in many groundwaters, a phenomenon which reflects the long nitrate travel times in the unsaturated and saturated zones. However, after controlling for daily rainfall patterns at baseline and follow-up, a more nuanced picture emerges (Table 3). The depth to water table is similar in the two areas (Table 1) and both areas comprise phonolites with a pyroclastic overlay and have similar shallow lateritic soils. This analysis therefore suggests that there may have been hazards that were unmeasured in our protocol, which were responsible for greater nitrate concentrations at baseline in Manyatta A and lower baseline nitrate concentrations in Migosi. Given that no well depth measurements were available from the baseline survey, it is also possible that changes in depth to water table and antecedent soil moisture conditions could have explained the changes between baseline and follow-up well water quality, although no long-term changes in water table depth had been observed by well owners. Whilst our protocol was constrained for this study by baseline data availability, a future protocol for identifying well water contamination hazards in informal urban settlements may therefore need further revision to better capture hazards such as leaky sewerage pipes and blocked storm drains, both via sanitary risk inspection and mapping of such hazards.

Given the public health significance of shallow well water contamination, in this study, we have therefore developed a protocol for ongoing monitoring of shallow well water quality that characterises the changing pattern of hazards such as pit latrines alongside water quality parameters. The protocol combines mapping with sanitary risk inspection and water quality testing. Elements of the protocol, such as mapping of contamination hazards and sanitary risk inspection, could potentially be taken up by community groups, following community-based monitoring initiatives in similar settings [23]. As noted above, there is also scope to further refine the protocol, for example by mapping other hazards such as uncollected refuse and blocked storm drains or including *in situ* rainfall measurement.

Since at least one well from the baseline was abandoned because of ingress of faecal contamination, the results here may under-estimate the increase in both water quality parameters reflecting contamination and sanitary risk. Our findings may also be affected by measurement error when assessing sanitary risk and testing water quality, although we attempted to minimize systematic bias by using the same field staff for both surveys and the same laboratory techniques wherever possible. For example, a lack of replicability in sanitary risk inspection would reduce the statistical power of our study to detect change in risk scores. Whilst such measurement errors may be averaged out when looking at groups of wells, they potentially inhibit our ability to assess the change in sanitary risk for individual wells, where their impact will likely be greater. We used a daily gridded rainfall time series to control for the short-term impacts of rainfall events on well water quality. However, several such data sets exist and their characteristics are known to differ, particularly in terms of representing extreme events that may be particularly associated with contamination [24]: this may have affected our findings.

More generally, given that evidence suggests shallow well water can be subject to transient contamination events [16], there is a need to sample such waters at greater frequencies than is possible with laborious manual testing techniques, such as the laboratory-based membrane filtration to detect thermotolerant coliforms in our study. Building on proof-of-concept remote monitoring of rural hand-pumps [25], there may be scope for example to monitor changes in well water quality via the use of solid state sensors measuring electro-conductivity and turbidity [26], alongside automated water-level metres [27].

Because of the availability of accessible baseline data, we have focussed here on two well-established urban neighbourhoods that have been part of the city of Kisumu for decades. However, the impact of urban population growth on shallow groundwater sources may also occur in emerging peri-urban settlements and around small towns, where high population growth is forecast [1]. Alongside more established urban settlements, there is thus also a need to understand the changing pattern of contamination risk in such emerging settlements. In this context, future urban form may be particularly influenced by current Chinese-led investment in road and public transport construction projects in much of sub-Saharan Africa, including Kisumu [28,29]. As longer distance travel becomes easier, such transportation upgrading may generate more dispersed urban settlements in the future, with potential implications for the future distribution of groundwater contamination hazards.

## 5. Conclusions 

Long-term groundwater quality monitoring efforts are generally focused on deep aquifers, but from a public health perspective, in sub-Saharan Africa, households are frequently exposed to potentially contaminated water from shallow aquifers. This case study suggests that in two well-established urban neighbourhoods with unplanned development, groundwater from hand-dug wells has persisted as a domestic water source for over a decade. However, with an intensification of settlement patterns and increased number of pit latrines, potential contamination hazards have also grown between 1999 and 2015. Whilst nitrate concentrations apparently fell between 2004 and 2014 despite the growing contamination risks, multivariate analysis suggests that short-term contamination events associated with high rainfall during sampling in 2004 may account for this apparent contradiction. With many urban households in sub-Saharan Africa relying on shallow groundwater and such use apparently persisting, there is a need to monitor and manage well water contamination risks and quality. In this study, we have developed a protocol for long-term monitoring of both contamination risks and well water quality. Given continued urban population growth in sub-Saharan Africa, this protocol requires further adaptation to the urban setting so that high risk water sources can be identified as part of a wider strategy to manage the public health risks from urban shallow wells.
